# Primary high-throughput screening of engineered phytases by online monitoring of the oxygen transfer rate of *Komagataella phaffii*

**DOI:** 10.1186/s12934-025-02806-w

**Published:** 2025-08-07

**Authors:** Sarah Luise Straaten, Marie Zöllner, Eva Forsten, Sekar Mayang W. Wahjudi, Anna Joëlle Ruff, Johanna Stotz, Ulrich Schwaneberg, Jørgen Barsett Magnus, Jochen Büchs

**Affiliations:** 1 Chair of Biochemical Engineering (AVT.BioVT), RWTH Aachen University, 52074 Aachen, Germany; 2 Chair of Biotechnology, RWTH Aachen University, 52074 Aachen, Germany

**Keywords:** Phytase screening, Recombinant protein production, Volumetric and specific enzyme activity, *Pichia pastoris*, *Komagataella phaffii*

## Abstract

**Background:**

Recombinant phytase production has recently gained increased recognition in phosphate recycling from phytate contained in plant-based side and waste streams. Until now, new phytase variants are evaluated at the end of the expression by standard offline screening procedures, where promising candidates with high activities and protein titers are identified. However, for large mutant libraries, this implies extensive laboratory work for a first screening of hundreds of clones. In this study, for the first time, two synergistic concepts for the primary screening of phytases were investigated.

**Results:**

The aim was to predict high recombinant protein producer strains as well as high volumetric activity phytase variants, based on the development of the respiratory activity over time of the host cell, in this case, *Komagataella phaffii (Pichia pastoris)*. In a first step, the metabolic burden was investigated by cultivating a clone library in YPD medium in a µTOM device. It was found that strains expressing medium or high protein concentrations show clear characteristics of an elevated level of metabolic burden during constitutive expression. However, a high protein concentration does not imply a high enzymatic activity. Therefore, in a second approach, the screening was adapted to screen for phytase variants with high volumetric activity. To do so, a modified Syn6 MES medium was developed, where phytic acid was used as the only phosphate source. Thereby, only clones secreting active phytase and generating free phosphate were able to grow, which was monitored via the oxygen transfer rate. A correlation between the offline measured volumetric phytase activity and µ_max_ was found. The clones were then ranked according to their online and offline performance and the results matched in 83% of the cases.

**Conclusion:**

Online monitoring of the oxygen transfer rates in 96-well plates allowed for the evaluation of the total protein concentration and the volumetric phytase activity already during the expression. Using these results, also the specific activity can be calculated. In the future, primary screening experiments of large enzyme mutant libraries can be conducted without offline activity assays, to identify promising candidates.

**Supplementary Information:**

The online version contains supplementary material available at 10.1186/s12934-025-02806-w.

## Background

Phosphate is an essential mineral for plants, animals, and humans and it is one of the main components of fertilizers [1, 2]. However, phosphate is a finite resource and is currently gained mostly by rock mining in a limited number of countries [3]. The expected lifetime of concentrated natural phosphate rock reservoirs is roughly estimated to be 300 years [4]. Also, inorganic phosphate is often radioactively contaminated [3, 5]. It has significant negative impacts on the environment, such as water eutrophication [6, 7]. Therefore, gaining phosphate from renewable resources is receiving more attention as an emerging part of a circular bioeconomy [8]. One approach is to use phytate, a primary storage form of phosphate in plants, stored in cereal grains, oilseeds, and legumes. It comprises 50–80% of the total phosphate in the plant [9]. Potential sources of phytate in plant materials are sugar beet slices, a side product in sugar production, or oilseed press cakes (rape or sunflower), also a side stream produced in oil manufacturing [10, 11]. Phytate is composed of an inositol ring and to each carbon atom, a phosphate group is bound. To release the phosphate groups from the inositol core, phytases, enzymes, which can hydrolyze the phosphate ester bonds, can be used [12, 13]. Currently, phytases are mainly used as a feed supplement for monogastric animals, such as poultry, swine, and fish [14]. Phytases reduce the anti-nutritional effect of phytic acid, a polyanionic chelating agent, and enhance the phosphate and mineral uptake from the plant feed material [15–17]. However, phytases are also found in human nutrition, food processing, non-food industrial products, and emerging applications like enzymatic phosphate recovery from renewable resources mentioned above [8, 15].

If phytases are used in industrial applications, often improved and more efficient phytase variants are needed. For instance, phytases in feed additives require higher thermal tolerance for feed pelleting processes (60–80 °C). In addition, a broad pH range and protease resistance of phytases are needed to withstand the gastrointestinal tract [18–20]. But regardless of the application, a high specific activity is always essential for economic feasibility. However, an ideal phytase enzyme with the above-mentioned properties may not be easily found in nature, which is why there is a continuous need to develop customized phytases for industrial requirements [18, 21]. Hence, the development in phytase engineering by directed evolution and rational design strategies is essential. One suitable expression host for engineered phytases is the methylotrophic yeast *Komagataella phaffii (Pichia pastoris)* [22, 23]. The advantages of *K. phaffii* as a production system for heterologous proteins are its abilities to grow to very high cell densities, to reach g/L concentrations of the protein of interest, and its secretory capabilities [24, 25]. In this study, the protein of interest, the *E. coli* AppA phytase (EC 3.1.3.26), is secreted by *K. phaffii* BSYBG11 (Mut^S^). State of the art for evaluating the engineered phytase variants is the quantification of the produced protein concentration [g/L] and the analysis of the volumetric enzyme activity [U/mL] by manual screening assays. The specific enzyme activity [U/mg] can be obtained by dividing the volumetric enzyme activity by the produced enzyme concentration (Eqs. 1 and 2).

Different approaches for simplifying the primary screening process have been investigated in the past. For example, the phenomenon of the metabolic burden has been exploited in *E. coli* and *K. phaffii* by evaluating the oxygen transfer rate (OTR) or the derivative of the scattered light [26–28]. In detail, the elevated protein production could be observed in the growth behavior of the host cell, as the limited metabolite resources of the cell are divided between biomass formation and protein synthesis [29]. In *E. coli*, it was demonstrated that a single amino acid exchange or even a silent codon exchange can directly influence the metabolic burden of the cell and protein production [30–32].

The limitation of evaluating the metabolic burden, visible in the OTR, is that only the total protein concentration and not the enzymatic activity, an equally important parameter, is recognized. In this study, for the first time, it is shown that the volumetric phytase activity can also be evaluated by online monitoring of the OTR, going beyond the estimation of only the total protein concentration. By dividing the volumetric activity [U/mL] by the produced enzyme concentration [g/L], also the specific activity [U/mg] can be calculated. The goal was to reduce the primary screening effort for large phytase mutant libraries. In literature, predominantly the methanol-induced p*AOX* promoter is used [33, 34], however, in this study, for a proof-of-principle, the GAP promoter was used for constitutive expression. As a first step, the concept of metabolic burden was investigated. Subsequently, a modified Syn6 MES medium for selective growth on phytic acid, for strains expressing highly active phytase variants, was developed. The oxygen transfer rate was used to monitor the metabolic activity online by conducting the cultivations in an in-house built µTOM device. In this device, the RAMOS technology is applied to 96-deep well microtiter plates (MTP) [35]. 96-well MTPs are the first-choice cultivation scale for screening applications because large numbers of clones can be cultivated in parallel [36, 37]. As the footprint of the µTOM device is not much larger than that of a standard MTP, a standard shaker tray can easily be loaded with e.g. 10 µTOM devices. In theory, using 96-well MTPs, 960 or 480 clones in duplicates could be tested in parallel. By combining the cultivation in 96-well plates and the online monitoring of the OTR, a high-throughput screening system for phytases could be established in this study.

## Materials and methods

### Strain and clone libraries

As a starting point for the clone libraries, the *K. phaffii* host strain BSYBG11 (Mut^S^), containing the plasmid pBSYAISIZ::appa-phytase-Zeocin, was used. The clone libraries (JE10, JE11, CB30, and IH17) were all generated with different mutagenesis strategies on the AppA *E. coli* phytase gene. In addition to the mutants, all libraries contained a clone with wildtype phytase and a clone with an empty vector, which were used as references. The CB30 library was generated using error-prone PCR with a concentration of 0.2 mM MnCl_2_. The other three libraries used site-saturation mutagenesis. For the JE10 library, degenerated nucleotides at position 216 were introduced, and for JE11 at position 267 of the gene. The IH17 library was generated with triple site-saturation mutagenesis. Therefore, mutations were introduced in three steps at positions 216, 267, and 305. Moreover, four model clones from these libraries were selected for preliminary tests of the medium conditions and workflow. Hereafter, they are referred to as CB30 (1), JE10 (1), JE11 (1) and empty vector. Number (1) indicates the position on the 96 well plate to identify the clone used. The expression was controlled by the constitutive GAP promoter in all clones.

### Media and solutions

All chemicals applied for media preparation were of analytical grade and purchased from Carl Roth GmbH (Karlsruhe, Germany), if not stated otherwise.

In this study, two media were used for cultivation: a complex medium, Yeast extract Peptone Dextrose (YPD) medium, and Syn6 MES minimal medium. YPD was prepared as a 2x concentrated stock solution. The stock solution is composed of 40 g/L peptone from meat (Charge: 022307073), 20 g/L Bacto^™^ yeast extract (Life Technologies Corporation, USA, Lot: 2352582) dissolved in water. In the final YPD medium glucose was added to 10 g/L. The composition of the Syn6 MES medium was as follows [26]: The basis Syn6 MES medium consists of 7.66 g/L (NH_4_)_2_SO_4_, 3.3 g/L KCl, 3.0 g/L MgSO_4_ × 7H_2_O, 0.3 g/L NaCl, 27.3 g/L 2-(N-morpholino) - ethane sulfonic acid, 4-morpholineethane -sulfonic acid (140 mM MES). The content of KH_2_PO_4_ in the experiments was either 0 or 1 g/L. In the modified medium, 0.11, 0.55, or 0.81 g/L phytic acid from sodium phytate was used as a phosphate source. The pH was adjusted to 6.0 with 1 M NaOH. The basic medium solution was sterilized via autoclaving (121 °C for 20 min). To obtain 1 L Syn6 MES medium, 940 mL basic medium is supplemented with 10 mL of 100 g/L CaCl_2_ (autoclaved), 10 mL of a 100 × micro-elements stock solution, 10 mL of a 100 × vitamin stock solution, 10 mL of a 100 × trace-elements solution, and the residual 20 mL are used to add a stock solution of the desired carbon source, in this case glucose was added to 10 g/L. The stock solutions had the following compositions: Micro-element stock solution: 6.65 g/L EDTA (ethylenediamine tetraacetic acid disodium sulfate), 6.65 g/L (NH_4_)_2_Fe(SO_4_)_2_ × 6H_2_O, 0.55 g/L CuSO_4_ × 5H_2_O, 2 g/L ZnSO_4_ × 7H_2_O and 2.65 g/L MnSO_4_ × H_2_O. Vitamin stock solution: 0.04 g/L d-biotin and 13.35 g/L thiamine chloride. The d-biotin was dissolved in 10 mL of a (1:1) mixture of 2-propanol and deionized water. Thiamin chloride was dissolved separately in 90 mL of deionized water. Afterward, the two solutions were mixed. Trace element stock solution: 0.065 g/L NiSO_4_ × 6H_2_O, 0.065 g/L CoCl_2_ × 6H_2_O, 0.065 g/L boric acid, 0.065 g/L KI, and 0.065 g/L Na_2_MoO_4_ × 2H_2_O. All stock solutions were filter sterilized. Zeocin was added to a final concentration of 25 µg/L.

### Cultivation methods

Both pre- and main cultures were cultivated in 96-deep square well MTPs (riplate SW, 2.5 ml, HJ-Bioanalytik GmbH, Erkelenz, Germany), sealed with a gas‐permeable sealing film (AeraSeal™-film, Excel Scientific, Inc. USA). The cultivation conditions for both pre- and main cultures were *T* = 30 °C, a shaking diameter of *d*_0_ = 50 mm, *n* = 350 rotations per minute (rpm) (Incubation shaker Climo Shaker, ISF1-X, Kuhner Shaker GmbH, Germany). These parameters ensured efficient mixing and oxygen transfer within the cultures. MTP cultivations were conducted using a working volume of *V*_*L*_= 600 µL, inoculated with 20 µL from either a cryo or pre-culture. To minimize evaporation, humidity in the incubation shaker was set to *H* = 85%. For the main cultures in YPD, the pre-culture was also prepared in YPD. For experiments in the modified Syn6 MES medium, independent of the phosphate concentration used in the main culture, the pre-culture was conducted with 0.135 g/L KH_2_PO_4_. All cultivations were online monitored in an in-house built µTOM device, where the oxygen transfer rate of each individual well was measured [35].

### Offline methods

Two different methods for protein quantification and one for the measurement of the volumetric activity were applied in this study. For establishing the correlation between the metabolic burden and the produced total protein concentration, the BCA assay was used. Using this easy-to-handle assay, only the total protein concentration was evaluated as a first step, not specifically the phytase concentration. Later, after a proof-of-principle was achieved, selected samples were measured by capillary electrophoresis to verify the correlation with the phytase concentration. This method is more specific, but also more expensive, and was, therefore, only used for a few samples. In the second screening approach, where a correlation between the OTR and the volumetric phytase activity was investigated, the 4-MUP assay was used. It is an established method to quickly measure the volumetric activity of phytases for large numbers of samples.

### BCA and 4-MUP assay

The Pierce™ BCA Protein Assay Kit (Thermo Scientific™, Inc. USA) was used to determine the total protein concentration in the supernatant. The phytase activity was measured by analyzing the supernatant with the 4-MUP assay [38]. In this assay, the produced phytases use the substrate 4-methylumbelliferyl phosphate (4-MUP) and cleave off the bound phosphate (Figure S1). The resulting product, 4-methylumbelliferone was excited with ultra-violet light at 360 nm and emitted fluorescence at 465 nm, which could be detected. The values obtained are in relative units since the relative activity is sufficient to identify promising clones from a large library in the primary screening. This method showed reliable and rather low standard deviations and may, therefore, be used in the first steps of a screening process [18, 38]. To perform the assay, all samples were diluted at a ratio of 1:10 in a 50 mM NaOAc buffer at pH 5.0. Next, 50 µL of a 1 mM solution of 4-MUP, dissolved in 50 mM NaOAc at pH 5.0, was added to 50 µL of the diluted supernatant. Fluorescence measurements were taken every minute for 15 min in the plate reader (BioTek™ Synergy™ Mx, BioTek Instruments, USA). A black MTP with a clear bottom (Microplate, 96 well, µCLEAR, Greiner Bio-One, Austria) was used, to prevent optical crosstalk with neighboring wells. The slope of the fluorescence increase was used to evaluate the phytase activity in the samples.

### Phytase quantification by capillary electrophoresis

The concentration of phytase in the culture supernatant was determined using the Bio-Rad Experion™ automated electrophoresis station (Hercules, USA). Prior to Experion™ analysis, samples were treated with Endo H for protein deglycosylation. The reaction was performed according to the manufacturer’s instructions (10 µg protein, 2 µl Endo H (1000 units) were added and the reaction was incubated for 18 h at 37 °C) (Promega, Mannheim, Germany). Subsequent Experion™ analysis was performed according to the Experion™ Pro260 analysis kit instructions, using bovine serum albumin (BSA) fraction V as an internal reference for protein quantification. BSA was added to the Endo H digestion at concentrations of 50–100 ng/µL (final concentration in the Endo H mixture).

### Conversion of produced total protein concentration and volumetric enzyme activity into specific enzyme activity

In the first screening approach, the metabolic burden (reduced µ_max_) is used to evaluate the produced total protein concentration [g/L]. For the produced enzyme concentration [g/L], it is assumed that

1 $$ \begin{gathered} produced\,enzyme\,concentration~\left[ {g/L} \right] \hfill \\ \quad = a*produced\,protein\,concentration[g/L] \hfill \\ \end{gathered} $$

where the factor $a$ is constant. From the measured total protein concentration and the measured phytase concentration, this factor was calculated to be a = 0.016 ± 0.0069 [-].

In the second screening approach, µ_max_ is used to evaluate the volumetric enzyme activity. Combining the results from both approaches, the specific activity can be calculated as follows:

$$volumetric\,\, enzyme\,\,activity\,\, \left[ {\frac{{\mu {\text{mol}}}}{{\min *{\text{mL}}}} = \frac{U}{{{\text{mL}}}}} \right]$$

2 $$\begin{gathered} specific \,enzyme \,activity \,\left[ {U/mg} \right] \hfill \\ \quad = \,\frac{{volumetric \,enzyme \,activity \,\left[ {U/mL} \right]}}{{produced \,enzyme \,concentration\left[ {mg/mL} \right]}} \hfill \\ \end{gathered} $$

## Results and discussion

### Medium and high recombinant phytase producer strains show characteristics of elevated metabolic burden

**Fig. 1:**
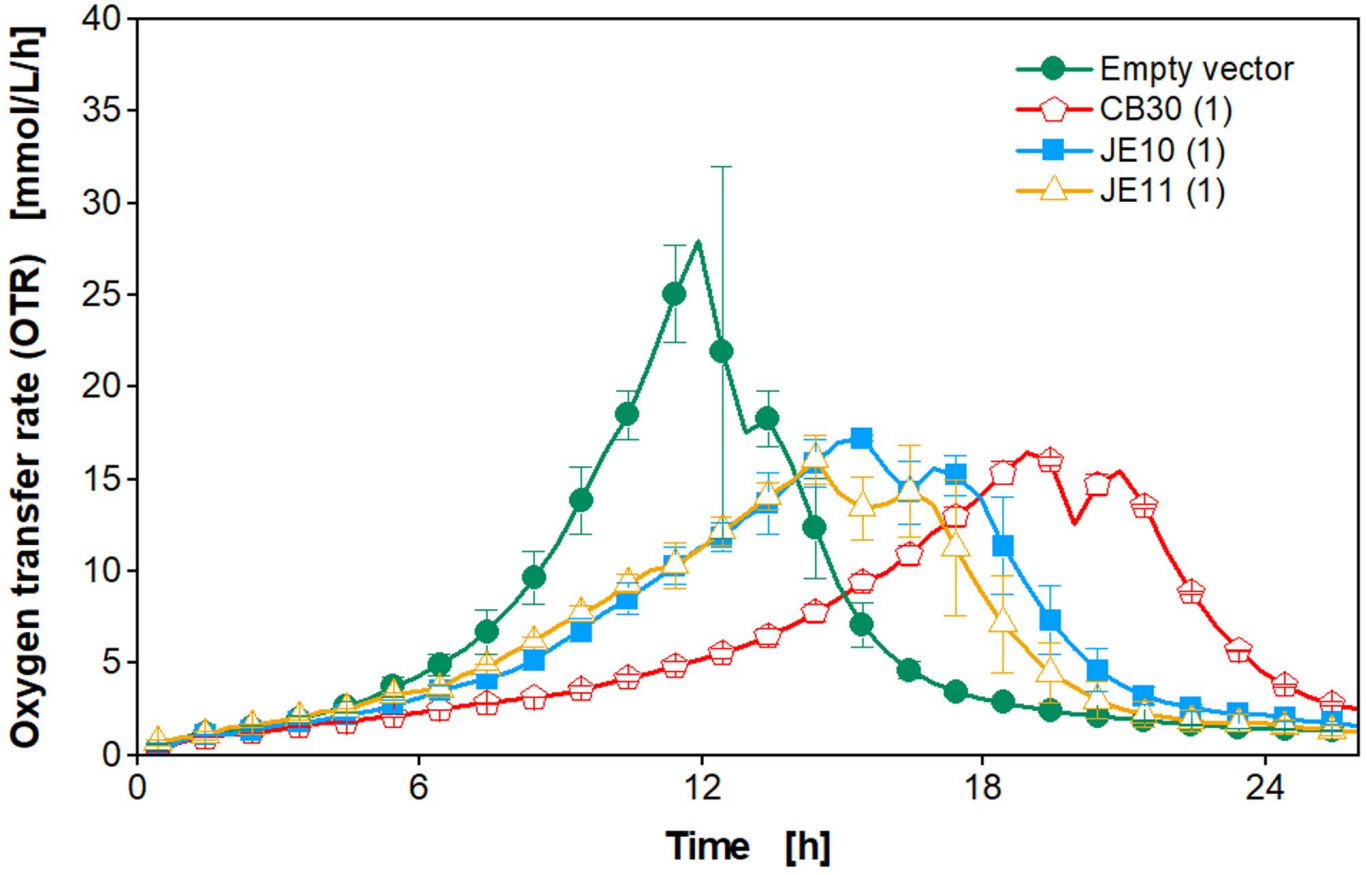
Cultivation of a clone library of a phytase-secreting *K. phaffii* strain. The cultivation was performed in YPD medium with 10 g/L glucose in a 96-square well MTP with 600 µL filling volume per well, operated at 350 rpm, 50 mm shaking diameter, and 30 °C. The oxygen transfer rate was monitored online in a µTOM device [35]. The data was obtained in duplicates and error bars represent minimal and maximal values. For clear data representation, four selected model clones are presented. Only every second measurement point over time is shown as a symbol. Data for the complete strain library can be found in Figure S2

As demonstrated in a previous study by Wollborn et al. (2022), the phenomenon of metabolic burden can be observed in *K. phaffii* by online monitoring of the oxygen transfer rate [26]. Whether this approach is also applicable to constitutive phytase expression, was tested by cultivating a clone library of a phytase-secreting *K. phaffii* strain in YPD medium in a µTOM device [35]. The aim was to identify the best-performing clones, producing the highest total protein concentration, by analyzing the growth rate until the first peak from the online measured OTR data (Fig. 1).

**Fig. 2:**
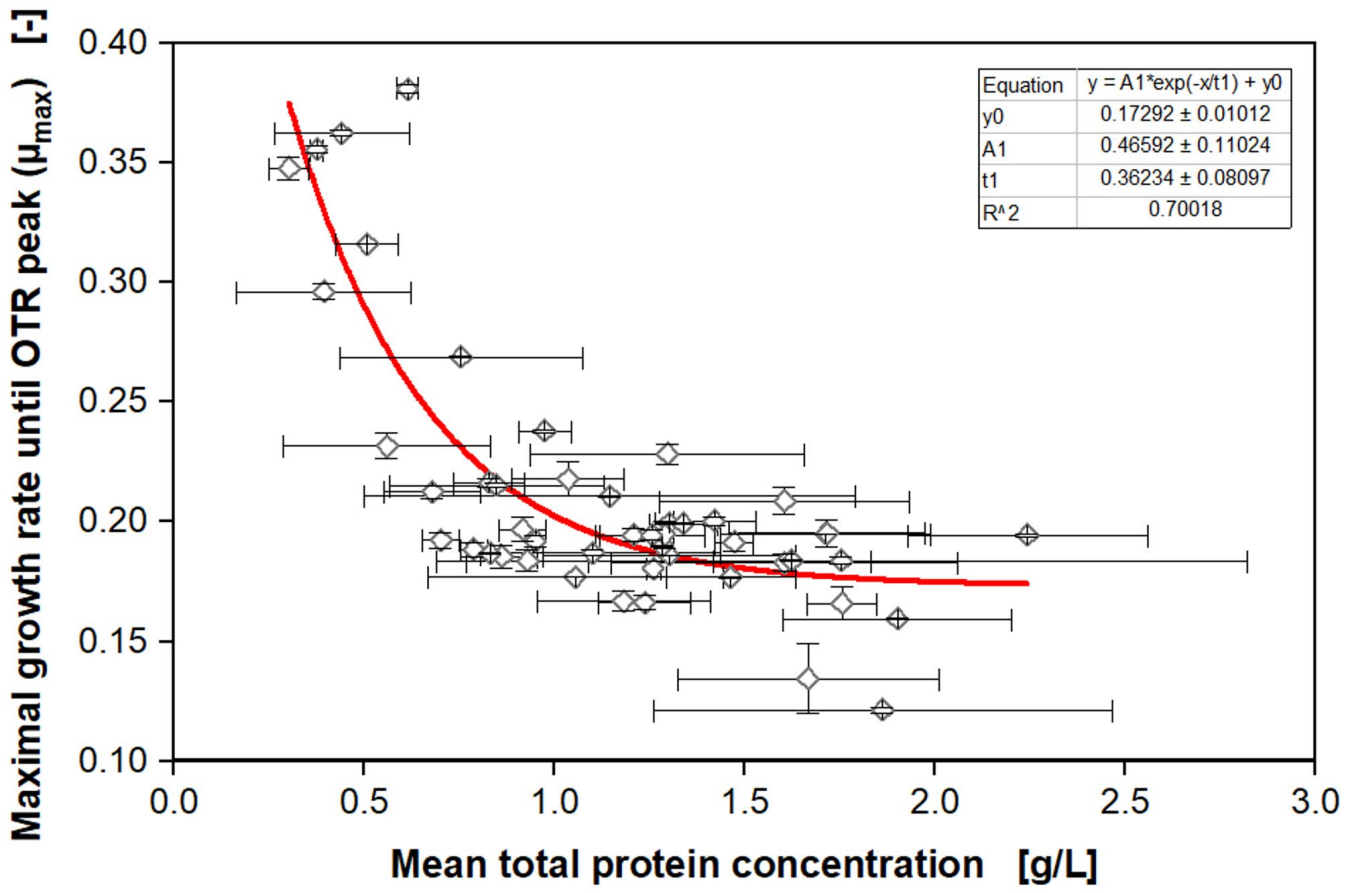
Correlation of calculated maximal growth rate (µ_max_) with offline measured protein concentration. The online measured oxygen transfer rates (OTR) in Fig. 1 and Figure S2 were used to calculate the maximal growth rate until the first OTR peak with an exponential fit, as shown in Figure S3. The mean total protein concentration was measured at the end of the cultivation after 26 h for each clone with a BCA assay. The mean of duplicates is shown for the offline protein concentrations and growth rates with error bars as minimal and maximal values. The cultivation was performed in YPD medium with 10 g/L glucose in a 96-square well MTP with 600 µL filling volume per well, operated at 350 rpm, 50 mm shaking diameter, and 30 °C in a µTOM device [35]

From Fig. 1, it becomes apparent that the growth of the four model clones from the screened library starts to differ around 4 h after inoculation. The empty vector without phytase expression reaches its maximum OTR of 27 mmol/L/h after the exponential phase at 12 h, whereas the other phytase-producing clones peak later at approximately 15 mmol/L/h at 14 and 19 h. All clones show a second peak shortly after the first, which possibly results from the formation and consumption of overflow metabolites, such as ethanol, acetate, and arabitol [39, 40]. The variation of growth profiles within the library (Fig. 1 and Figure S2) indicates that increased phytase expression in this biological system redirects the carbon flux from biomass to protein production, thus, resulting in a decreased OTR [26]. To verify this hypothesis, the mean protein concentration for each of the 48 clones was determined offline with a BCA assay, while the mean growth rate until the first OTR peak was calculated, as described in Figure S3 (shown for one example). For quantification of the metabolic burden, µ_mean_ ~ µ_max_ was assumed. This is justified because the lag-phase is negligible, there is no initial substrate excess inhibition, and it can be assumed that [S] > > K_S_ (S = glucose) for the time interval used for fitting. Analyzing the parameters µ_max_ and mean protein concentration (Fig. 2), a negative exponential correlation becomes apparent, which is visualized by the red curve. Clones with a high maximal growth rate (µ_max_ > 0.25) exhibited a low mean protein concentration of less than 1 g/L, whereas growth rates lower than µ_max_ = 0.25 correlate with higher protein concentrations of up to 2.3 g/L. This observation indicates that the concept of metabolic burden applies to this phytase expression system. As the constitutive GAP promoter is used, expression starts from the beginning of the cultivation. Wollborn et al. (2022) already successfully demonstrated the applicability of the metabolic burden concept in *K. phaffii* during the methanol-induced expression of GFP, where a titer of 18 mg/L seemed to be sufficient to cause metabolic burden [26]. In the clone library used in this study, phytase concentrations of 10–30 mg/L were reached and led to a reduced growth rate (Fig. 2 and Figure S4). The measured total protein concentrations by BCA in the range of 0.5–2.5 g/L were significantly higher than the determined phytase concentrations (Fig. 2 and Figure S4). The differences in titers between the two methods might be explained by secreted endogenous proteins that contribute to the measured total protein concentration [41–43]. Nevertheless, the correlation of both, the total protein and phytase concentrations, with µ_max_ was very similar and confirmed the concept of this screening approach. The main limitation, however, is that medium producers cannot be distinguished from high ones, as the error bars from the manually performed, conventional BCA assay are very high. On the contrary, the OTR profiles are very reproducible, as demonstrated by the small vertical error bars in Fig. 2.

It can be concluded that this experimental setup is suitable for the primary screening of phytase-producing strains. Poor producers, regarding the total protein concentration, can be clearly identified and excluded from further screening steps. Also, promising clones can be detected with significantly less laboratory work compared to conventional approaches. However, a high total protein concentration does not necessarily correlate with high specific or volumetric activity in the context of mutant screening. This parameter, though, as well as the specific activity, is a decisive factor in industrial applications. To address this challenge, a second, completely different, approach was investigated, where a new cultivation medium for screening the best-performing clones, regarding volumetric phytase activity, was developed.

### Clones secreting highly active phytases can be identified by growth on phytic acid

When a large mutant library is generated by e.g. error-prone PCR, not only improved variants of the enzyme are obtained but also inactive ones. These variants, though, are still analyzed offline via activity assays after the cultivation [18, 39]. To make the primary analysis via activity assays for these unsuitable clones obsolete, in the following, a new screening method, based on the growth on phytic acid, monitored by the OTR, is presented.

**Fig. 3:**
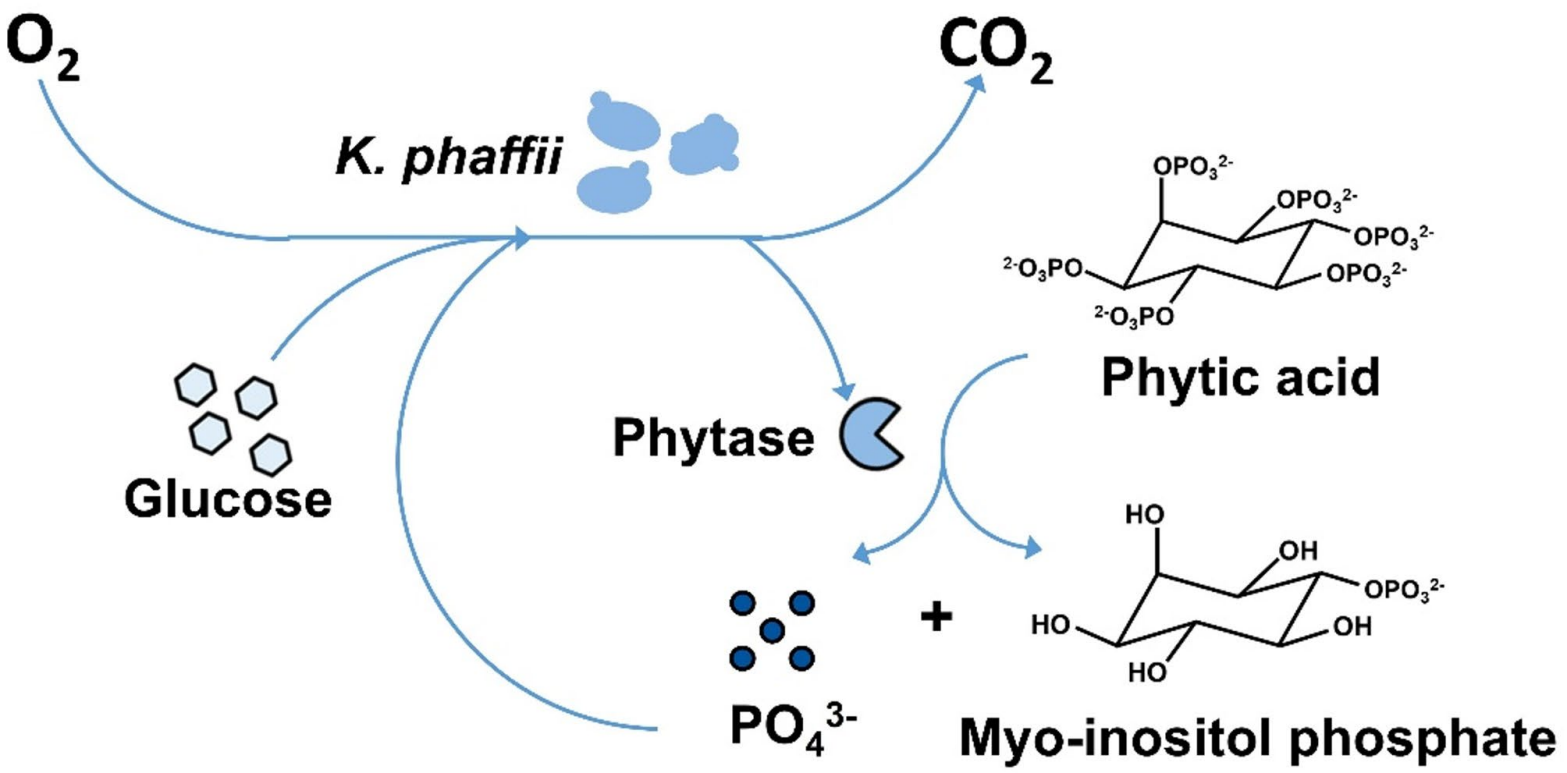
Schematic illustration of the phytase screening principle in modified Syn6 MES medium. Phytic acid is used as the only source of phosphate in the modified Syn6 MES medium. If the expression host, *K. phaffii*, secretes an active variant of the phytase enzyme, phosphate units from the phytic acid molecule are cleaved to generate *myo*-inositol phosphate and free phosphate. The full conversion to *myo*-inositol phosphate is hypothetical

The minimal Syn6 MES medium has been shown to be suitable for heterologous protein expression in *K. phaffii* and other yeasts [39, 44]. In contrast to YPD medium, it is a buffered system. The pH in YPD medium was observed to drift up to 8.34 ± 0.05 (Table S1), which is unfavorable for growth. In the Syn6 MES medium, the pH could be kept stable at around 5.64 ± 0.01 until the end of the cultivation. A higher biomass was also observed (OD_600_ of 11.25 ± 0.28 in Syn6 MES compared to 8.23 ± 0.53 in YPD) (Table S1). Moreover, using a defined medium is essential for growth-based activity screening assays, especially as a defined amount of phosphate is required in the following screening approach. Therefore, Syn6 MES medium was used as a starting point for the development of a new cultivation medium, in which only clones secreting an active phytase variant can grow. The new screening approach is illustrated in Fig. 3.

**Fig. 4:**
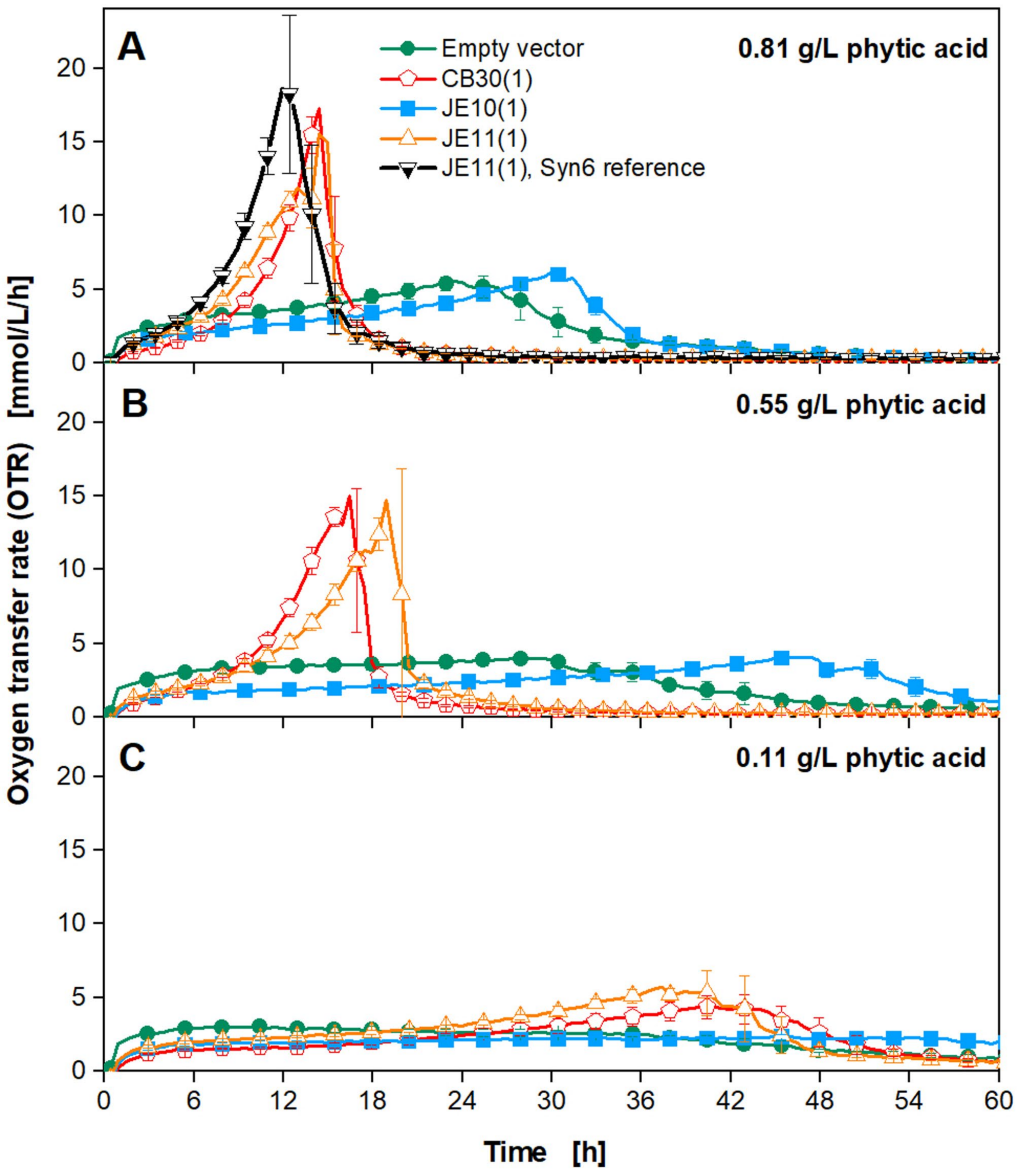
Cultivation of selected model clones of phytase-secreting *K. phaffii* strains. The cultivation was carried out in the modified Syn6 MES medium with 140 mM MES buffer (pH = 6.0), 10 g/L glucose, and (**A**) 0.81 g/L, (**B**) 0.55 g/L and (**C**) 0.11 g/L phytic acid as the only phosphate source. A 96-square well MTP was used with 600 µL filling volume per well, operated at 350 rpm, 50 mm shaking diameter, and 30 °C. The oxygen transfer rate was monitored online in a µTOM device [35]. The black curve in (**A**) represents a reference cultivation of the JE11 (1) clone in the standard Syn6 MES medium with 1 g/L KH_2_PO_4_, for comparison. The data was obtained in triplicates and error bars represent the standard deviations. For clear data representation, only every third or fifth measurement point over time is shown as a symbol

The new medium does not contain inorganic phosphate. Phytic acid is the sole phosphate source. The phosphate groups, though, are bound to the phytic acid molecules and, thus, only *K. phaffii* clones secreting active phytase variants can release the phosphate. It is unknown how many phosphate units from the phytic acid molecule each mutated phytase can cleave. Together with glucose and other medium components, the phosphate is taken up and used for growth by *K. phaffii*. By generating more biomass, more phytase is secreted as well, which then generates more free phosphate that is available for growth. The growth of each *K. phaffii* clone was monitored online by measuring the respiratory activity in a µTOM device [35]. Glucose is used as the sole carbon source. It was found that *myo*-inositol cannot be metabolized by *K. phaffii* (Figure S5). To demonstrate a proof-of-principle, four pre-selected model clones from different mutant libraries (Table 1), were cultivated in the new screening medium. Three different concentrations of phytic acid were tested: 0.81 g/L (Fig. 4A), which is the same molar concentration of phosphate as 1 g/L KH_2_PO_4_, and reduced concentrations of 0.55 (Fig. 4B) and 0.11 g/L (Fig. 4C). From expression experiments in YPD medium, the performance of the selected model clones is known and shown in Table 1.

**Table 1:** Characteristics of model clones in YPD medium in screening experiments.

Model clone	Mutagenesis strategy	Total protein concentration (g/L)	Volumetric phytase activity (U/mL)	Specific phytase activity (U/mg)
Empty vector	–	Low (0.51)	None (0)	None (0)
JE11 (1)	site-saturation mut. pos. 267	Medium (1.05)	High (3.95)	High (242.01)
JE10 (1)	site-saturation mut. pos. 216	Medium (0.86)	Low (0.1)	Low (7.48)
CB30 (1)	epPCR 0.2 mM MnCl_2_	High (1.90)	High (3.37)	Medium (114.10)

Each model clone originates from a clone library generated with a different mutagenesis strategy (mut.: mutagenesis, pos.: nucleotide position, epPCR: error-prone PCR). (1) stands for the position on the 96-well plate. From each library, the clone on position 1 was used as a model clone. The specific activity was calculated according to Eqs. 1 and 2. The four model clones exhibit different growth behaviors in the new screening medium as shown in Fig. 4A and B, where the phytic acid concentration is 0.55–0.81 g/L. In Fig. 4A, clones CB30 (1) and JE11 (1) show exponential growth only slightly delayed, compared to the black reference curve, which is a cultivation in standard Syn6 MES medium (containing inorganic phosphate). CB30 (1) and JE11 (1) show an OTR peak around 17 mmol/L/h at 15 h, whereas the reference has its OTR maximum at 12 h. Clones JE10 (1) and the empty vector only reach an OTR of 6 and 5 mmol/L/h after 32 and 24 h, respectively. For the fast-growing clones, a sharp drop in the OTR after the peak is visible, while for the slower-growing ones, a triangle shape can be observed. This indicates a secondary substrate limitation [45–47], in this case most likely by phosphate. The black reference curve demonstrates unlimited growth. As clones CB30 (1) and JE11 (1) perform similarly to the reference, it can be concluded that the phytase mutant secreted by those clones is indeed active and able to cleave off phosphate groups from the phytic acid molecule and use it for growth on glucose. In contrast, the empty vector, in theory, cannot enzymatically cleave any phosphate. However, a small increase in the OTR can be observed. This indicates that either phosphate from the pre-culture (conducted with 0.135 g/L KH_2_PO_4_) could be stored internally in the cell and used for later growth under phosphate depletion [48] or that some of the phosphate groups are cleaved by autolysis. As all cultures of the empty vector were inoculated with the same preculture, the amount of internally stored phosphate, and, therefore, the OTR course of the empty vector, should be the same in Fig. 4A–C. As this is not the case, the hypothesis that some phosphate is available through autolysis is supported. Via HPLC, the batch of phytic acid used was analyzed. It was found that no free phosphates are present (data not shown). Thus, possible autolysis of phytic acid by phosphatases secreted by *K. phaffii* under phosphate starvation would be a suitable explanation [49]. Though, as the growth curve of the empty vector can be clearly distinguished from the model clones secreting active phytase variants, the autolysis does not impede the functionality of this screening approach. It should be noted, that the results from Fig. 4A perfectly match the expected results from Table 1, where clones CB30 (1) and JE11 (1) are indicated as well-performing while the empty vector and JE10 (1) are not able to cleave phosphates from phytic acid.

As the clone JE10 (1) has an extended growth compared to the empty vector, it can be assumed that in addition to the autolysis, some free phosphate is generated by the secreted phytase of this clone. This is coherent with the previously determined characteristics of the model clones (Table 1), where JE10 (1) only has a low volumetric activity and medium protein concentrations, compared to the other model clones. Using 0.81 g/L phytic acid (Fig. 4A), the two model clones with high or medium volumetric phytase activity (CB30 (1) and JE11 (1)) can already be distinguished from the clones with none or low volumetric activity. However, as the two better-performing clones lie very closely together, the total concentration of phytic acid used in the medium might be too high and the concentration of available phosphate is not sufficiently limiting the growth. The concentration of 0.11 g/L has been shown by Capusoni et al. to enable growth for various yeast strains [50]. Therefore, 0.55 g/L was chosen as an intermediate concentration. When only 0.55 g/L phytic acid is used (compare Fig. 4B), the growth of clones CB30 (1) and JE11 (1) differs significantly in the exponential phase. It is important to note that with these experimental conditions, only clones with phytase variants that use inositol hexaphosphate (InsP_6_ or phytic acid) are selected for growth. Other variants that use e.g. inositol tetra- or triphosphate may not be able to grow. However, in the literature, it is reported that the appA *E. coli* phytase is able to break down InsP_5_ into the following intermediates (InsP_4_-InsP_1_) after the initial, almost complete conversion of phytic acid to InsP_5_ [51]. This is consistent with the data from Fig. 4A, where the clones CB30 (1) and JE11 (1) show very similar OTR courses compared to the reference curve, as both experimental conditions, 1 g/L KH_2_PO_4_ and 0.81 g/L phytic acid, theoretically supply the same concentration of phosphate.

Interestingly, for all four model clones, the OTR peak reaches lower values with 0.55 g/L, compared to 0.81 g/L phytic acid, including the empty vector. In a study by Naghdi et al., it was observed that a phytase from *E. coli* does not follow the Michaelis Menten kinetic, but rather exhibits a sigmoidal saturation curve [52]. 0.81 g/L (1.23 mM) phytic acid is theoretically already in the saturated area of the kinetic (Figure S6), while 0.55 g/L (0.83 mM) is in the transition area. 0.11 g/L (0.17 mM) lies in the linear area but corresponds to very low activities (ca. 0.0002 µmol/min). This is coherent with the data from this study, where phytase variants can be distinguished with 0.55 g/L phytic acid, while 0.11 g/L (Fig. 4C) is too low, and only minimal growth is observed. Furthermore, both, the produced total protein concentration and the level of volumetric phytase activity, influence the concentration of free phosphate available in the medium. As CB30 (1) shows faster growth until the first OTR peak, it would be ranked as a better phytase variant. However, by looking at the online OTR data, it cannot be distinguished to what extent the total protein concentration and the volumetric activity individually contribute to the free phosphate and, thus, the growth profile. However, with 0.55 g/L both peaks could be nicely separated and with only 0.11 g/L (Fig. 4C) almost no growth was visible. Therefore 0.55 g/L of phytic acid was chosen as a suitable concentration for the screening medium, to achieve a sufficient differentiation of the growth rates.

Comparing both screening approaches discussed above, it can be concluded that a flat OTR course over time is desired when determining the produced total protein concentration [g/L] in the first approach. In contrast, a steep OTR course is favorable, when the volumetric activity [U/mL] is evaluated in the second approach. When the results of both methods are divided, it is possible to obtain the specific activity [U/mg], which is an equally important parameter (Eqs. 1 and 2).

**Fig. 5:**
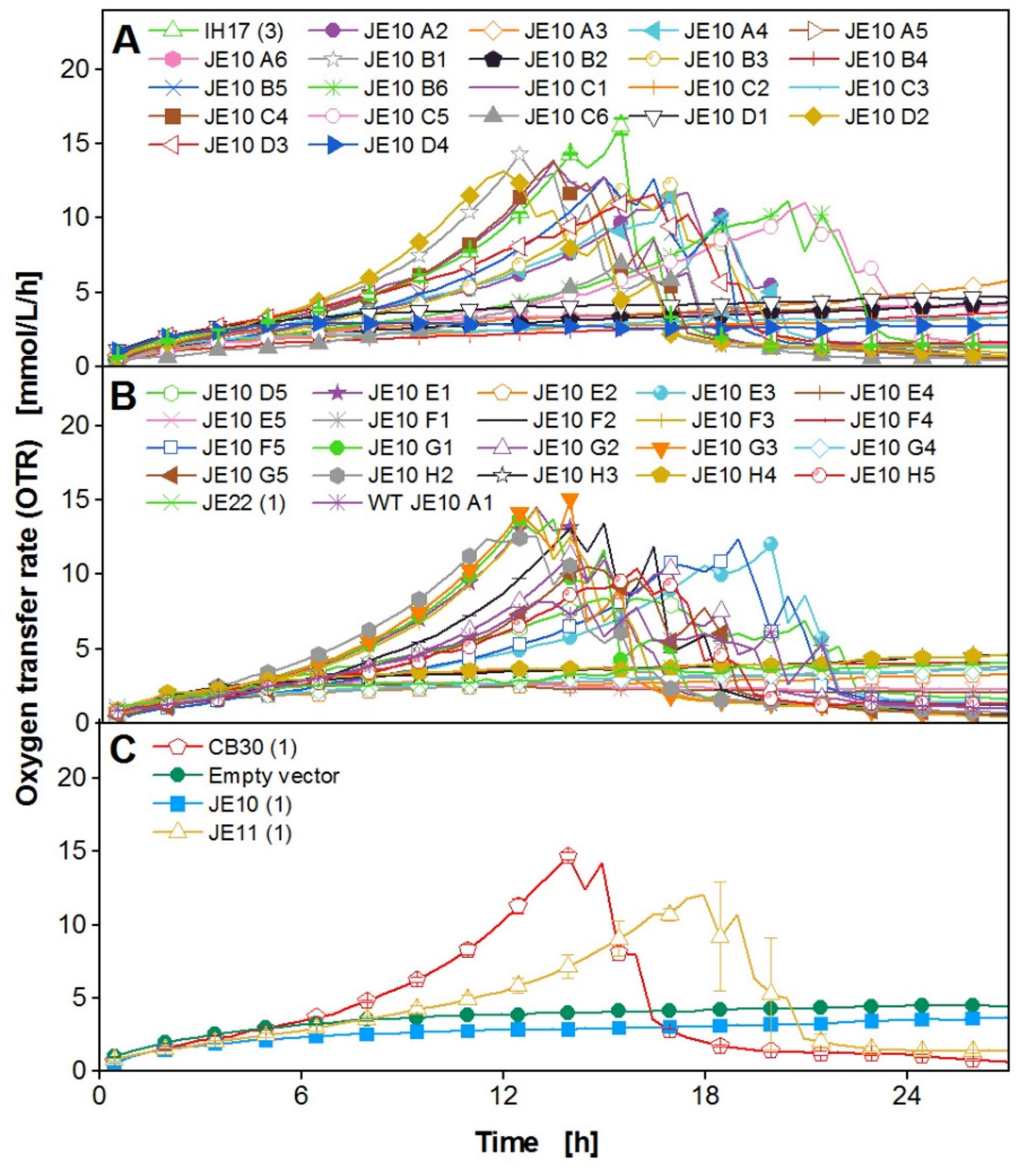
Cultivation of a clone library (JE10) of a phytase-secreting *K. phaffii* strain. The cultivation was performed in the modified Syn6 MES medium with 140 mM MES buffer (pH = 6.0), 10 g/L glucose, and 0.55 g/L phytic acid in a 96-square well MTP with 600 µL filling volume per well, operated at 350 rpm, 50 mm shaking diameter, and 30 °C. The oxygen transfer rate was monitored online in a µTOM device [35]. **A** The mean OTR data for 22 of the 48 tested clones is shown without error bars for clarity. **B** The mean OTR data for another 22 of the 48 tested clones is shown without error bars for clarity. **C** The OTR data of the four selected model clones is displayed. The data was obtained in duplicates and error bars represent minimal and maximal values. For clear data representation, only every third measurement point over time is shown as a symbol

After finding a suitable phytic acid concentration and a proof-of-principle, the next step was to evaluate the screening approach with a larger strain library. To do so, the libraries JE10 (Fig. 5A, B) and IH17 (Figure S7) were cultivated in duplicates in 96-square well plates in a µTOM device [35]. The model clones were also cultivated on the same microtiter plate to check for reproducibility (Fig. 5C).

**Fig. 6:**
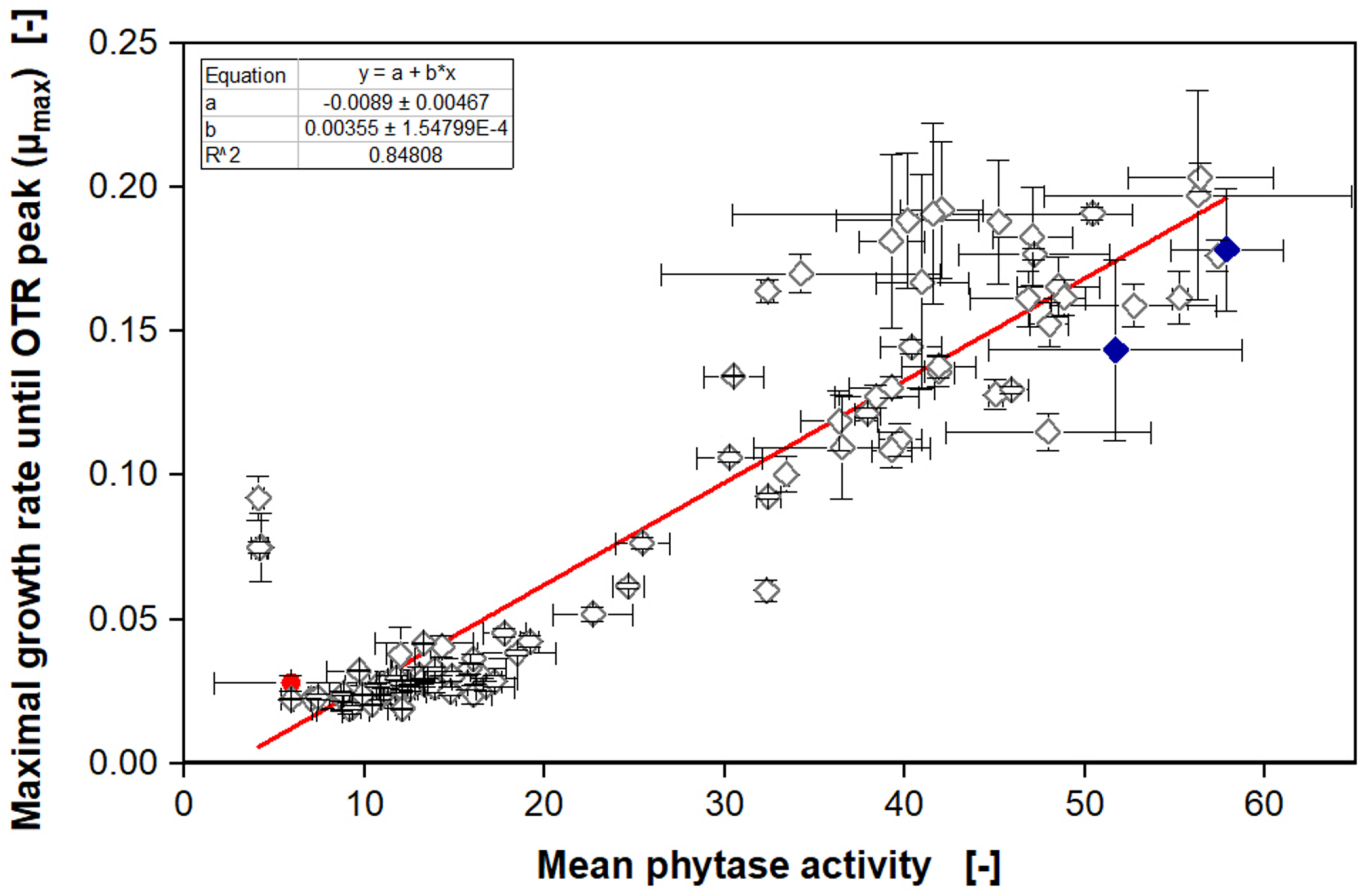
Correlation of calculated maximal growth rate (µ_max_) with offline measured phytase activity. The online measured oxygen transfer rates (OTR) from 96 clones (Fig. 5 and Figure S7) were used to calculate the maximal growth rate until the first OTR peak with an exponential fit, as shown in Figure S3. The mean phytase activity was measured at the end of the cultivation after 26 h for each clone with a 4-MUP assay. The mean of duplicates is shown for the offline phytase activities and growth rates with error bars as minimal and maximal values. The cultivation was performed in the modified Syn6 MES medium with 140 mM MES buffer (pH = 6.0), 10 g/L glucose, and 0.55 g/L phytic acid in a 96-square well MTP with 600 µL filling volume per well, operated at 350 rpm, 50 mm shaking diameter, and 30 °C in a µTOM device [35]. A linear fit was performed, to visualize the correlation between the online-measured maximal growth rate and the offline-measured volumetric phytase activity. The empty vector clones are marked as filled red circles and the wild type clones, which contain the non-modified phytase gene, are marked as filled blue diamonds

Figure 5 demonstrates that a wide spectrum of OTR courses was generated in one experiment. As before, some clones exhibit high growth rates and OTR values up to 15 mmol/L/h after 14 h, while for others the OTR only increases up to 3 to 5 mmol/L/h. The model clones in Fig. 5C show very good reproducibility, compared to the previous experiment (Fig. 4B) (note that the x-axis is scaled differently). In both experiments, the OTR of CB30 (1) increases up to 15 mmol/L/h after 15 h, whereas the first OTR peak of JE11 (1) occurs at 18 h. After successfully cultivating the two libraries in the µTOM (JE10, IH17, and model clones) and ensuring reproducibility, the OTR data was evaluated, and maximal growth rates until the first OTR peak were calculated (Figure S3 shows one example). The correlation of the calculated maximal growth rates from the online data and the offline measured phytase activity (determined via 4-MUP assay) is presented in Fig. 6.

**Fig. 7:**
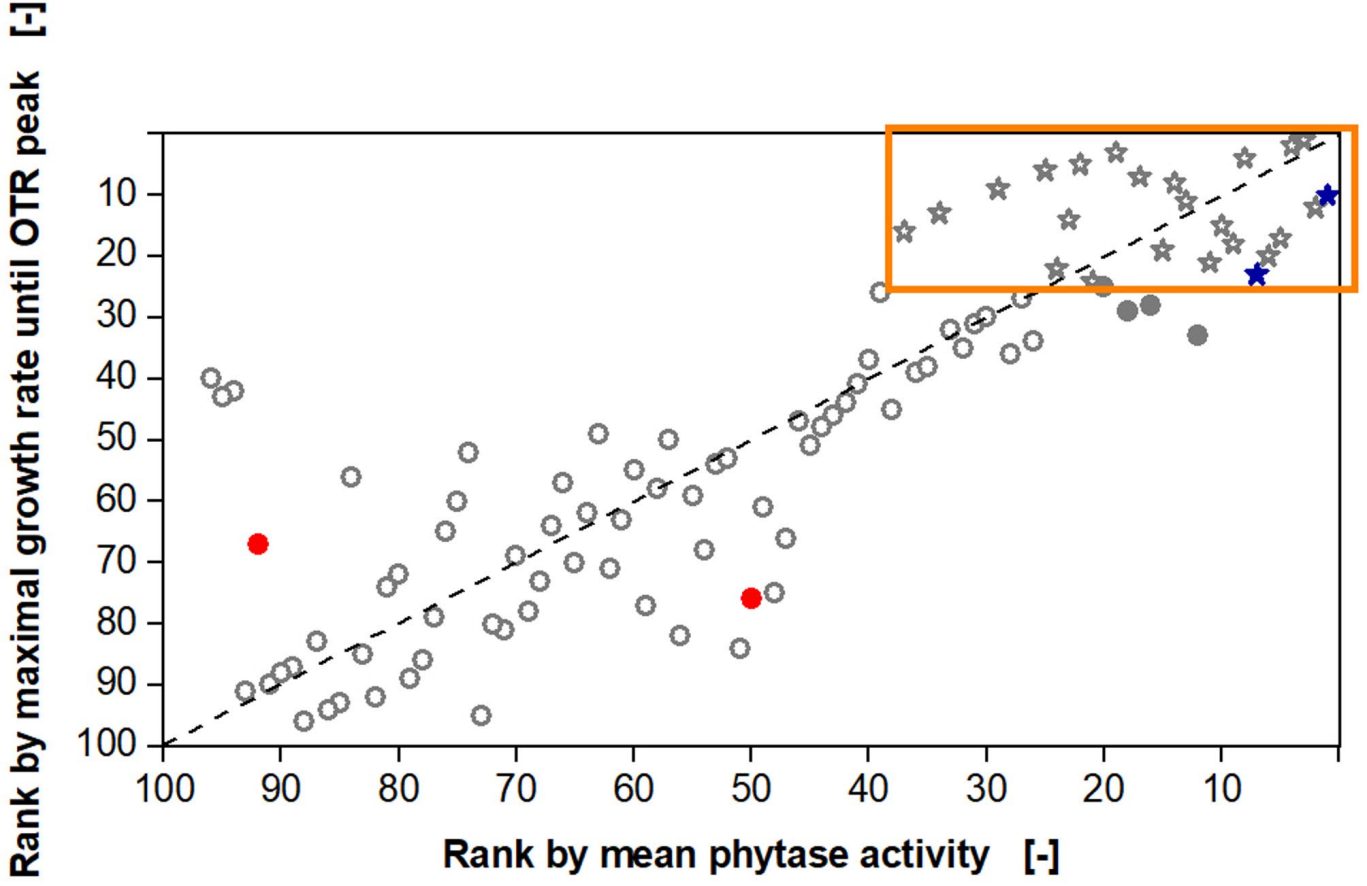
Ranking of clone performance by the maximal growth rate and offline measured phytase activity. First, all 96 tested clones were ranked according to their maximal growth rate until the first oxygen transfer rate (OTR) peak and second, according to their offline measured phytase activity (data shown in Fig. 6). The clone with the highest maximal growth rate would be ranked as number 1 on the y-axis and the clone with the highest phytase activity as number 1 on the x-axis. The orange box and the star symbols mark the top 25% of clones, evaluated by the growth rate from the online measured OTR (24 clones). The empty vector clones are marked as filled red circles and the wild type clones as filled blue stars. Clones, which would be lost in this screening round, are marked as filled grey circles

The online and offline data from all 96 clones investigated are depicted in Fig. 6 and maximal growth rates of 0.01 to 0.2 until the first OTR peak were observed. A linear fit was performed for visualization. The phytase activity values from the 4-MUP assay are in arbitrary and relative units but contain the relevant information for the primary evaluation of phytase performance. Figure 6 reveals that clones with none or very low volumetric activity exhibit very low growth rates of 0.05 or less, except for two outliers. At higher phytase activities, the correlation is not distinct anymore. Clones with a volumetric phytase activity higher than 30, exhibit growth rates in a wider range from 0.09 to 0.2. Nevertheless, a roughly linear correlation can be recognized. As expected, the empty vector shows no activity and is found in the lower left corner, where a dense cloud of data points covers the second empty vector data point (not visible in Fig. 6). The wild type phytase clones from the two experiments showed relatively high volumetric phytase activity. From this result, it can already be concluded that the mutagenesis approaches, applied for the two libraries, led to only a few improved phytase variants. However, the overall goal of this experiment was to identify the best phytase variants regarding their volumetric activity, only by online monitoring the OTR during growth on the newly developed screening medium. Therefore, a ranking was performed, based on both online and offline data and is shown in Fig. 7.

In Fig. 7, both, the x and y-axis, were reversed so that the top performing phytase variants (ranked as number one in online and offline data) are located in the top right corner. The orange box and the star-shaped symbols mark 25% of the best clones based on the online measured OTR data (24 clones out of 96). In a scenario, where only online monitoring of the OTR during growth on phytic acid is conducted as a first primary screening, the best 25% would be chosen for further offline activity measurements. By performing a primary screening with online monitoring of the OTR, in this case, 20 out of the 24 true best active variants were identified, which is ~ 83%. In this context, “true” refers to the offline measured activity. Also, the number of candidates from a clone library that must be analyzed manually via activity assays, can be drastically reduced. Obviously, when performing such a primary screening, some well-performing phytase variants will be lost. In this experiment, 4 out of the 24 best performers would be lost (marked as filled grey circles). However, 75% fewer clones would have to be analyzed manually, which immensely reduces time, resources, and costs. In total, a primary screening, in which some improved variants may be lost, would be justified, as the benefits of the reduced manual workload outweigh the limitations.

## Conclusion

A high-throughput screening system for recombinantly expressed phytase in *K. phaffii* was successfully established. As a first step, the concept of metabolic burden was exploited to evaluate clones, based on their produced total protein concentration. Clones were cultivated in YPD medium in the µTOM device, and the maximal growth rate (µ_max_) was calculated based on the slope of the OTR until the first peak. Afterwards, the metabolic burden was assessed by comparing the calculated µ_max_ from online data to the offline measured protein concentrations via BCA assay. A low µ_max_ indicated a stronger metabolic burden and, thus, protein formation. A correlation between µ_max_ and the offline measured protein concentration was found. However, only clones with low protein concentrations could be clearly identified, based on their high µ_max_. Medium and good producers could not be easily distinguished by this method. However, this first approach was suitable to exclude non-promising producer strains. In a second approach, a primary screening for phytase variants with high volumetric activity was successfully developed. The Syn6 MES medium was modified into a screening medium, where phytic acid, the substrate for phytases, was the only phosphate source. Thus, only clones secreting an active phytase variant were able to grow. This metabolic activity was online monitored by measuring the OTR. Again, the slope of the OTR until the first peak was calculated and compared to offline measured phytase activity. Though, this time, a high maximal growth rate correlated to a high volumetric phytase activity. Online and offline measurement results were ranked, and a linear correlation was found. When taking the best 24 clones, based on the online measured growth rate, which would be clones worth to further analyzing thoroughly, it was found that 20 out of the 24 selected clones are also in the best 25% of the clones ranked by phytase activity. Thus, the rankings of the offline and online methods match in ~ 83% of the cases. All in all, in this study, two different synergistic concepts were applied to screen phytase mutant libraries, with regard to the produced total protein concentration and the volumetric activity of the enzyme. Phytase variants that are able to specifically cleave lower inositol phosphates are of high interest, as phosphate utilization from phytic acid is further increased. In the future, the screening approach for the volumetric activity could be applied to select strains, that secret phytases able to only use lower inositol phosphates as a substrate. Enzymatic preparation of these inositol phosphates would be necessary, to then use these as a phosphate source in a screening experiment corresponding to the second approach. This way, the activity screening method could be adapted for a variety of substrates of phytases. In conclusion, it was demonstrated that the oxygen transfer rate is a powerful tool to simplify the primary screening of large mutant libraries, where not only the total protein concentration is evaluated, but also the volumetric enzyme activity. By combining both screening approaches and dividing the volumetric enzyme activity [U/mL] by the produced enzyme concentration [g/L], it is also possible to obtain the specific activity [U/mg], which is often the most interesting parameter.

## Supplementary Information

Additional file 1.

## Data Availability

The datasets supporting the conclusions of this article are included within the paper and the additional file (Figures S1– S7, Table S1). The datasets used and analyzed during the study are available from the corresponding author upon reasonable request.

## Abbreviations

a: Constant factor
epPCR: Error-prone PCR
K_S_: Half-velocity constant
MES: 2-(N-morpholino) ethanesulfonic acid
µ_max_: Maximum specific growth rate
µ_mean_: Mean growth rate
µTOM: Micro(µ)‐scale Transfer rate Online Measurement
MTP: Microtiter plate
4-MU: 4‐Methylumbelliferone
4-MUP: 4‐Methylumbelliferyl phosphate
OTR: Oxygen transfer rate
PCR: Polymerase chain reaction
[S]: Concentration of the limiting substrate
YPD: Yeast extract peptone dextrose
