# Supplementary material for: Primary high-throughput screening of engineered phytases by online monitoring of the oxygen transfer rate of Komagataella phaffii
Source: Microb Cell Fact. 2025 Aug 7;24:180. doi: 10.1186/s12934-025-02806-w (PMC12330186; doi:10.1186/s12934-025-02806-w)
Supplement: Supplementary file 1 — Additional file 1. [file 12934_2025_2806_MOESM1_ESM.docx]

# Supplementary information


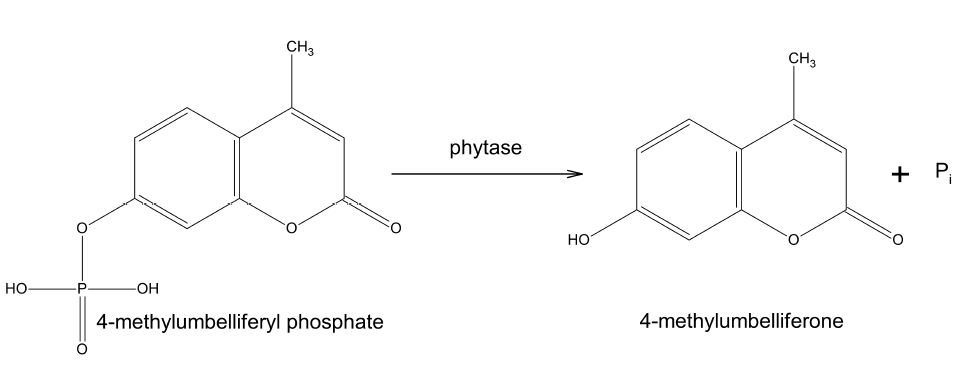


**Figure S1: Dephosphorylation of 4‐methylumbelliferyl phosphate (4‐MUP) to 4‐methyl‐ umbelliferone (4‐MU) catalyzed by alkaline phosphatase (ALP).**


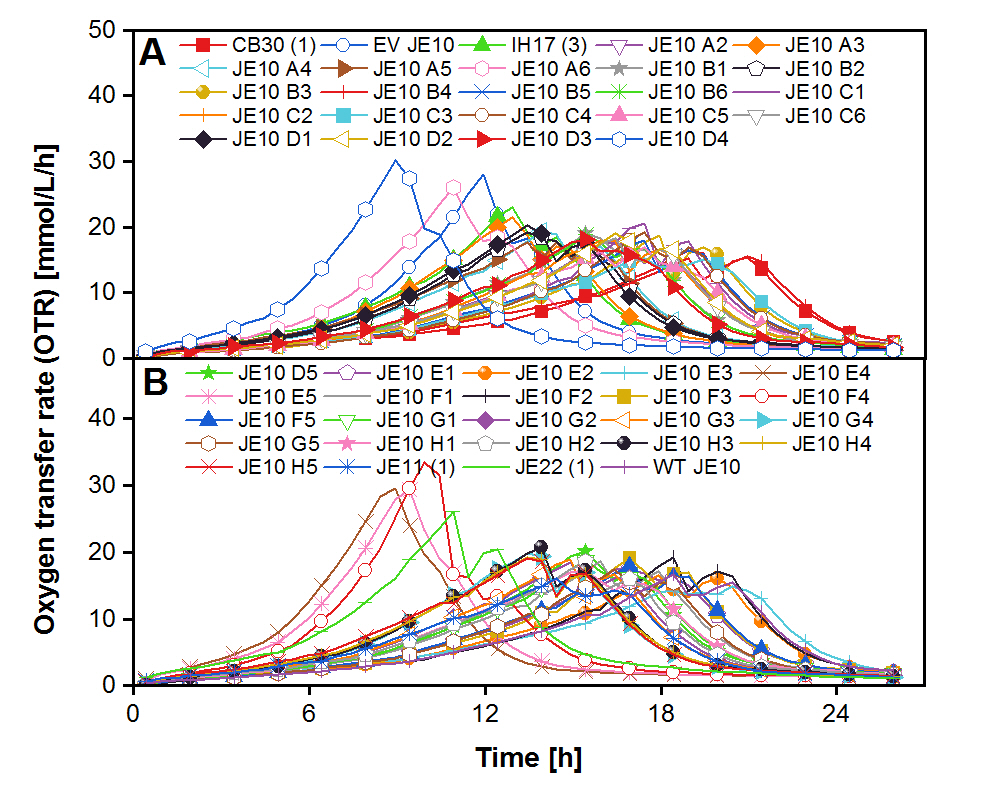


**Figure S2: Cultivation of a clone library (JE10) of a phytase-secreting *K. phaffii* strain.** The cultivation of 48 clones was performed in YPD medium with 10 g/L glucose in a 96-square well MTP with 600 µL filling volume per well, operated at 350 rpm, 50 mm shaking diameter, and 30 °C. The oxygen transfer rate was monitored online in a µTOM device (35). The data was obtained in duplicates but is shown without errors for clarity. For clear data representation, only every second measurement point over time is shown as a symbol, the first 24 clones are shown in (**A**) and the remaining ones in (**B**).


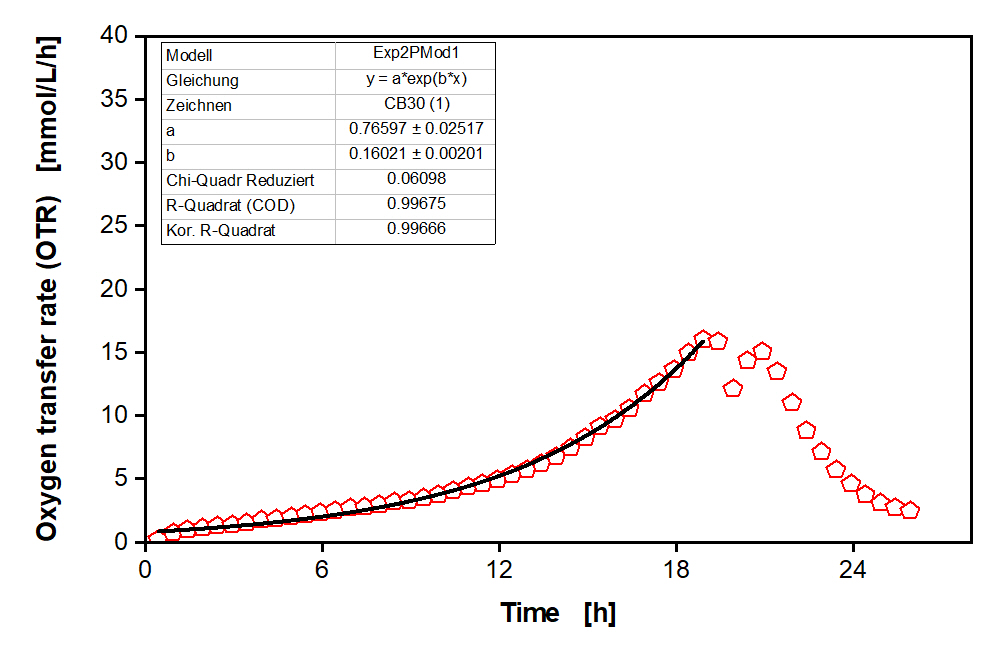


**Figure S3: Calculation of the maximal growth rate from the online measured oxygen transfer rate (OTR).** An exponential fit with the equation $OTR={OTR}_{0}\cdot e^{(\mu_{max} \cdot t)}$ was performed until the first peak in the oxygen transfer rate. Exemplarily, the fit is shown for the model clone CB30(1), cultivated in YPD medium with 10 g/L glucose in a 96-square well MTP with 600 µL filling volume per well, operated at 350 rpm, 50 mm shaking diameter, and 30 °C in a µTOM device (35). The fit for each growth curve of a clone library was performed with a MATLAB script.


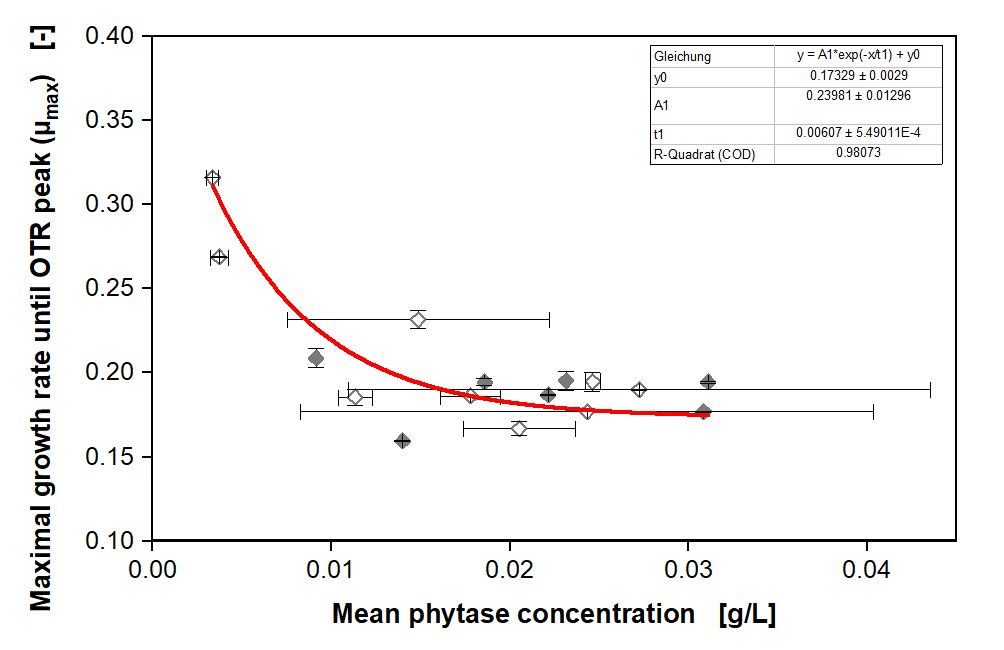


**Figure S4: Correlation of the calculated maximal growth rate with offline measured phytase concentration by capillary electrophoresis.** The online measured oxygen transfer rates (OTR) in Figure 1 and Figure S2 were used to calculate the maximal growth rate until the first OTR peak with an exponential fit, as shown in Figure S3. In addition to the offline BCA assay, selected samples at the end of the cultivation were also analyzed in a capillary electrophoresis, to determine the phytase concentration. The mean of duplicates is shown for the maximal growth rates and offline phytase concentrations with error bars as minimal and maximal values. Samples marked in dark grey were only measured as a single replicate in the capillary electrophoresis. The cultivation was performed in YPD medium with 10 g/L glucose in a 96-square well MTP with 600 µL filling volume per well, operated at 350 rpm, 50 mm shaking diameter, and 30 °C in a µTOM device (35).


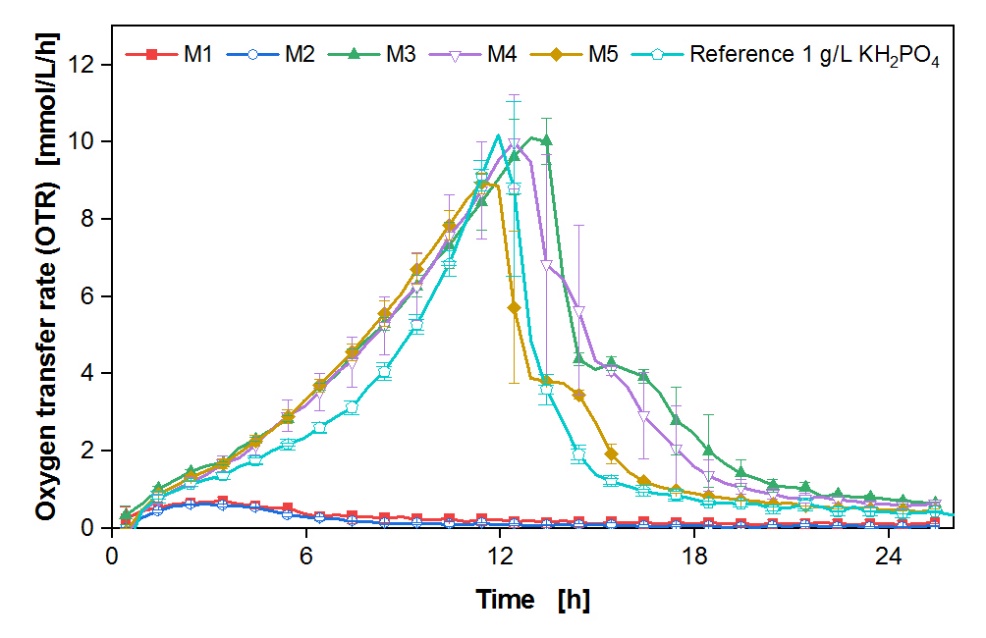


**Figure S5: Cultivation of model clone CB30(1) in different media variations to test for metabolization of *myo*-inositol.** Clone CB30(1) was cultivated in the modified Syn6 MES medium with 140 mM MES buffer (pH=6.0) in a 96-square well MTP with 600 µL filling volume per well, operated at 350 rpm, 50 mm shaking diameter, and 30 °C in a µTOM device (35). Following variations of the medium were tested: **M1**: 0 g/L glucose, 1.6 g/L phytic acid, 0 g/L *myo*-inositol, 0 g/L KH_2_PO_4_; **M2**: 0 g/L glucose, 0 g/L phytic acid, 4 g/L *myo*-inositol; **M3**: 10 g/L glucose, 0 g/L phytic acid, 0.22 g/L *myo*-inositol; **M4**: 9.15 g/L glucose, 0 g/L phytic acid, 1.09 g/L *myo*-inositol; **M5**: 8 g/L glucose, 0 g/L phytic acid, 2.18 g/L myo-inositol. M2-M5 each contained 1 g/L KH_2_PO_4_. For M3, M4, and M5 the *myo*-inositol content was adapted to keep the total amount of carbon constant. The mean of triplicates is shown with error bars as standard deviations.


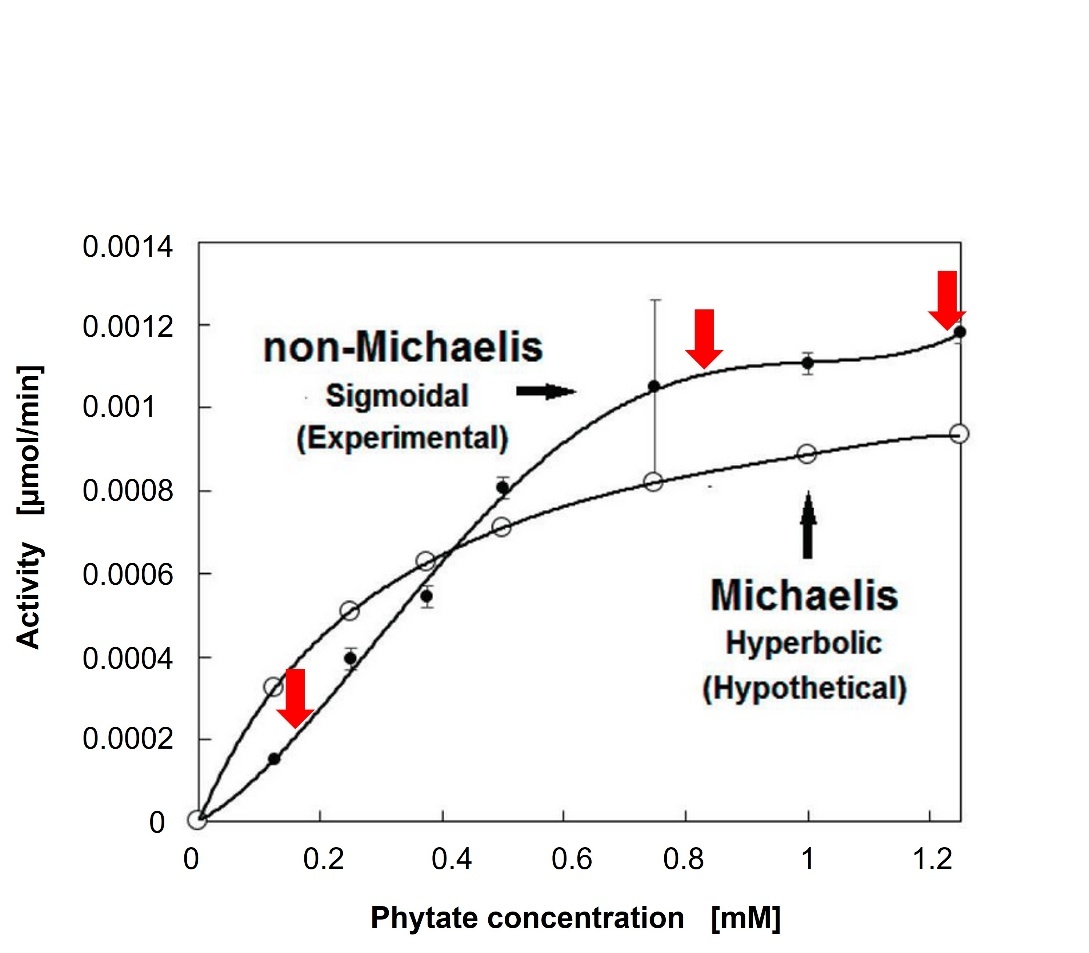


**Figure S6: Experimental kinetic of *E. coli* phytase, published by Naghdi et al. (52).** The phytase activity is shown over the molar concentration of the substrate phytate (phytic acid). The concentrations used in this study are marked with red arrows (1.23 mM, 0.83 mM, and 0.17 mM).


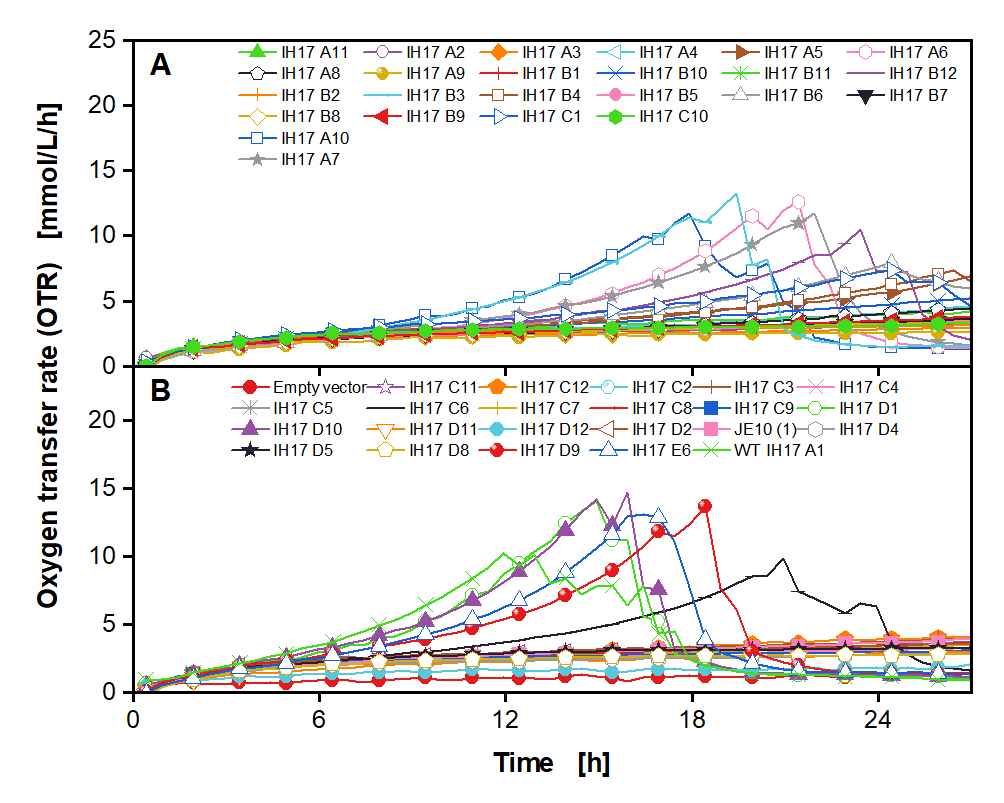


**Figure S7: Cultivation of a clone library (IH17) of a phytase-secreting *K. phaffii* strain.** The cultivation was performed in the modified Syn6 MES medium with 140 mM MES buffer (pH=6.0), 10 g/L glucose, and 0.55 g/L phytic acid in a 96-square well MTP with 600 µL filling volume per well, operated at 350 rpm, 50 mm shaking diameter and 30 °C. The oxygen transfer rate was monitored online in a µTOM device (35). The data was obtained in duplicates but is shown without errors for clarity. For clear data representation, only every third measurement point over time is shown as a symbol. The first 24 clones are shown in (**A**) and the remaining ones in (**B**).

**Table S1: Offline data from shake flask experiments.** The mean of two samples is shown for final OD_600_ and pH values for the empty vector.

| Medium | Mean final OD_600_ | Mean final pH |
| --- | --- | --- |
| YPD | 8.23 ± 0.53 | 8.34 ± 0.05 |
| Syn6 MES | 11.25 ± 0.28 | 5.64 ± 0.01 |
